# LONELINESS IN OLDER ADULTS LIVING IN PUERTO RICO DURING THE COVID-19 PANDEMIC: AN UNDEREXAMINED PUBLIC HEALTH CONCERN

**DOI:** 10.1093/geroni/igad104.1994

**Published:** 2023-12-21

**Authors:** David Camacho, Matthew Morgan, Denise Burnette

**Affiliations:** University of Illinois, Chicago, Chicago, Illinois, United States; Virginia Commonwealth University, Richmond, Virginia, United States; Virginia Commonwealth University, Richmond, Virginia, United States

## Abstract

Loneliness is a critical public health problem, yet it is under-examined among older adults in Puerto Rico. We explored sociodemographic and health-related risks for loneliness using data from a Knowledge, Attitudes and Practices COVID-19 study of Older Adults in Puerto Rico study. We conducted telephone and face-to-face interviews (January - December 2021) with a nonprobability sample of adults aged 60+ in Puerto Rico (n= 213). Data collection included measures of objective and subjective physical and mental health. We used generalized linear regression analyses to explore main effects of salient demographic and health factors on loneliness. Missing data were treated with Multiple Imputation. Prevalence of loneliness (3-item TILS) was 36.3% . Widowhood (β=.832, SE=.38, p=.029), low household income (<$12,500; β=.691, SE=.23, p=.003), psychological distress (β=.141, SE=.03, p<.001) were positively associated with greater loneliness. Living alone (β=-.664, SE=.27, p=.015) and higher sense of community (β=-.067, SE=.01, p<.001) were negatively associated with loneliness. Pandemic specific challenges (e.g., someone close diagnosed or hospitalized with COVID-19) and other common predictors (e.g., age, female, self-rated health) were not significant. We consider that contextual (e.g., outmigration, under-resourced aging and healthcare infrastructure) and cultural factors (e.g., familism) may help shape predictors and their effects on loneliness. Mainland US and Puerto Rican researchers and practitioners should collaborate on examining loneliness narratives, their impact on health, and implementation of culturally appropriate and contextually feasible interventions in Puerto Rico.

